# Aldosterone Antagonists Reduce the Risk of Cardiovascular Mortality in Dialysis Patients: A Meta-Analysis

**DOI:** 10.1155/2019/1925243

**Published:** 2019-03-03

**Authors:** Yan Li, Na Xie, Min Liang

**Affiliations:** Division of Nephrology, Nanfang Hospital, Southern Medical University, Guangzhou, Guangdong, China

## Abstract

*Background and Purpose. *Cardiovascular disease is the major cause of death in dialysis patients. Although aldosterone antagonists were considered a treatment for severe heart failure patients to reduce cardiac mortality, whether treating patients undergoing maintenance dialysis with aldosterone antagonists could reduce the risk of cardiocerebrovascular (CCV) remains unclear. We aim to systematically assess the efficacy and tolerability of the addition of aldosterone antagonists to conventional therapy in patients undergoing maintenance dialysis.* Materials and Methods. *We searched PubMed, EMBASE, the Cochrane Library, the Chinese Biomedical Literature Database (CBM), and the China National Knowledge Infrastructure (CNKI) for relevant articles. The primary endpoint of interest was CCV mortality. The secondary endpoints were all-cause mortality, left ventricular mass index (LVMI), and left ventricular ejection fraction (LVEF). Publication bias was evaluated using funnel plots and Egger's test. The meta-analysis was performed using Review Manager software version 5.3.* Results.* This analysis included 10 randomized controlled trials (RCTs) with 1172 total chronic dialysis patients. The use of aldosterone antagonists in the dialysis population resulted in a marked reduction in CCV mortality (RR 0.42, 95% CI 0.26-0.65, P=0.0002) and all-cause mortality (RR0.46, 95%CI 0.32-0.66, P<0.0001). The LVEF was improved by treatment with aldosterone antagonists (WMD 6.35%, P<0.00001). Moreover, aldosterone antagonists decreased the LVMI (WMD -8.69 g/m^2^, P=0.0006), whereas aldosterone antagonists increased the occurrence of hyperkalemia (RR1.70, 95%CI 1-2.88, P=0.05) and gynecomastia (RR 8.01, 95% CI 2.44- 26.27, P=0.0006).* Conclusions.* Addition of aldosterone antagonists to conventional treatment in chronic dialysis patients may reduce CCV mortality, improve cardiac function, and simultaneously decrease LVMI.

## 1. Introduction

In patients on dialysis, the prevalence of cardiac disease is relatively high, with an increased risk of cardiac disease and mortality [1]. Cardiovascular and renal functions are mostly regulated by the renin-angiotensin-aldosterone system (RAAS). Abnormal activation of the RAAS results in the development of hypertension, cardiovascular events, and chronic kidney disease (CKD) [2, 3]. Therefore, targeting the RAAS is an effective approach. Currently, interruption of the RAAS with angiotensin-converting enzyme (ACE) inhibitors and angiotensin receptor blockers (ARBs) has become a leading strategy in slowing the progression of kidney disease. However, combined therapy was associated with a higher risk of the composite events of dialysis, elevated creatinine level, and death. Otherwise, incomplete blockade of the renin-angiotensin cascade is frequently observed in patients chronically treated with ACEI or ARBs, a phenomenon known as “aldosterone escape” [4]. This phenomenon leads to the progression of cardiac and renal disease [5]. Although the mechanisms of the aldosterone escape phenomenon are not clear, targeting aldosterone with aldosterone antagonist may offer additional cardiovascular and renal protection against adverse and cardiovascular events [6–8].

In fact, in 1999, Pitt et al. reported a randomized aldosterone antagonist evaluation study (RALES) and concluded that blockading the aldosterone receptors using aldosterone antagonists could substantially reduce the risk of both morbidity and death in patients with severe heart failure [9]. Since then, many groups have carried out animal and clinical trials and have determined that aldosterone antagonists exhibit cardioprotective properties in many diseases [10, 11]. Recently, aldosterone antagonists have been reported to exert beneficial, prognostically significant cardiovascular effects in CKD patients [12].

Actually, although chronic dialysis patients have a high risk for cardiac problems, we rarely use aldosterone antagonists to reduce cardiac mortality in this situation, mainly due to the high risk of hyperkalemia, while some reports and reviews have shown that receiving a low dose of aldosterone antagonist and restricting potassium intake could prevent hyperkalemia. Additionally, the dialysis itself could remove the excessive potassium [13]. Using aldosterone antagonists in dialysis patients appears to be an excellent method to reduce cardiac mortality. There have been a number of clinical studies regarding aldosterone antagonists on cardiac mortality in dialysis patients, whereas there has been no systemic review to analyze the exact efficacy of aldosterone antagonists in this specific group of patients. Therefore, we undertook a meta-analysis to assess the effect of aldosterone antagonists on cardiocerebrovascular (CCV) mortality and cardiac function in cardiocerebrovascular (CKD) patients undergoing maintenance dialysis.

## 2. Methods

This report followed the preferred reporting items for systematic reviews and meta-analyses (PRISMA) guidelines (S1) [14].

### 2.1. Search Strategy

We searched the electronic databases PubMed, EMBASE, the Cochrane Library, the Chinese Biomedical Literature Database (CBM), and the China National Knowledge Infrastructure (CNKI) for studies published up to September 2018. The search terms that we used were aldosterone antagonists and dialysis. Details of the search method are outlined in S2. Reference lists of articles and reviews were hand searched for additional studies. There were no limitations placed on the publication language for the search. Two reviewers (Yan Li and Na Xie) independently assessed all the relevant studies. Any disagreements were resolved by discussion.

### 2.2. Selection Criteria

Two reviewers (Yan Li and Na Xie) independently screened the databases for titles and abstracts. If either reviewer felt a title or abstract met the study eligibility criteria, the full text of the study was retrieved. The eligibility criteria for inclusion in the review were as follows: the studies were randomized controlled trials (RCTs) that compared the effects of aldosterone antagonists with placebo or no treatment on CCV mortality, all-cause mortality, and cardiac function in adults (>18 years) undergoing maintenance dialysis without language restriction. Additionally, the studies included a minimum treatment duration of 12 weeks. When multiple publications reported the same or overlapping data, we used the most recent or largest population.

### 2.3. Exclusion Criteria

Exclusion criteria were as follows: nonrandomized, single-arm clinical trials; studies about adults with CKD stage 1-4; studies of less than 12-week duration. Studies were also excluded if they did not provide sufficient data.

### 2.4. Data Extraction

Data were independently extracted from the individual studies by two reviewers. The extraction forms of the two reviewers were compared. Disagreements were resolved through discussion to reach a consensus and, if necessary, a third reviewer was consulted. We extracted the following information: participants' dialysis method, CCV mortality, all-cause mortality, baseline and follow-up left ventricular mass index (LVMI), left ventricular ejection fraction (LVEF), type and dosage of aldosterone antagonist and control intervention, the incidence of serious hyperkalemia (defined as a serum potassium concentration of at least 6.0 mmol per liter or discontinuation of treatment because of hyperkalemia), and nonphysiologic gynecomastia. CCV mortality was defined as death from a CCV event [14]. CCV events included new occurrence or exacerbation of heart failure that was not improved by water removal through dialysis (clinical symptoms together with left ventricular dysfunction by echocardiography according to the American Heart Association [AHA]/American College of Cardiology [ACC] guidelines), malignant ventricular arrhythmias (ventricular fibrillation or sustained ventricular tachycardia), new or recurrent acute myocardial infarction (changes on electrocardiography [ECG] and biomarkers for myocardial infarction), new occurrence or exacerbation of angina pectoris (ECG change corresponding to chest symptoms and coronary angiography showing >75% stenosis according to AHA/ACC guidelines), dissecting aneurysm of the aorta (diagnosed by imaging techniques), stroke (diagnosed by computed tomography [CT] and/or magnetic resonance imaging [MRI]), new or recurrent transient ischemic attack (TIA) (diagnosed by CT and/or MRI and sudden onset of neurological deficit persisting for <24 h), and SCD [15]. We withdrew the information using the Get-data software, as some studies described the change in LVMI and LVEF using figures instead of using digital means.

### 2.5. Quality Assessment

The Cochrane risk-of-bias tool was used to assess the methodological quality of each study. The risk of bias in each eligible trial was independently assessed by two reviewers (Yan Li and Na Xie). Publication bias was evaluated using funnel plots and Egger's test.

### 2.6. Statistical Analysis

We used Review Manager (RevMan) software version 5.3 for the analysis. As the CCV mortality, all-cause mortality was dichotomous; these data were analyzed using a risk ratio (RR) with 95% confidence intervals (CIs), whereas the continuous variables (changing from baseline to follow-up) were analyzed using weighted mean differences (WMD). Statistical heterogeneity was measured using the I^2^ statistic; pooled analyses were calculated using fixed effect models if I^2^ < 50%, whereas random effects models were applied in cases of I^2^ ≥ 50%. Publication bias was evaluated using funnel plots and Egger's test. Sensitivity analysis (excluding one study at a time) was performed to determine the stability of the overall treatment effects. A P value of ≤0.05 for any test or model was considered to be statistically significant.

## 3. Results

### 3.1. Study Selection and Quality Assessment

**Figure 1:**
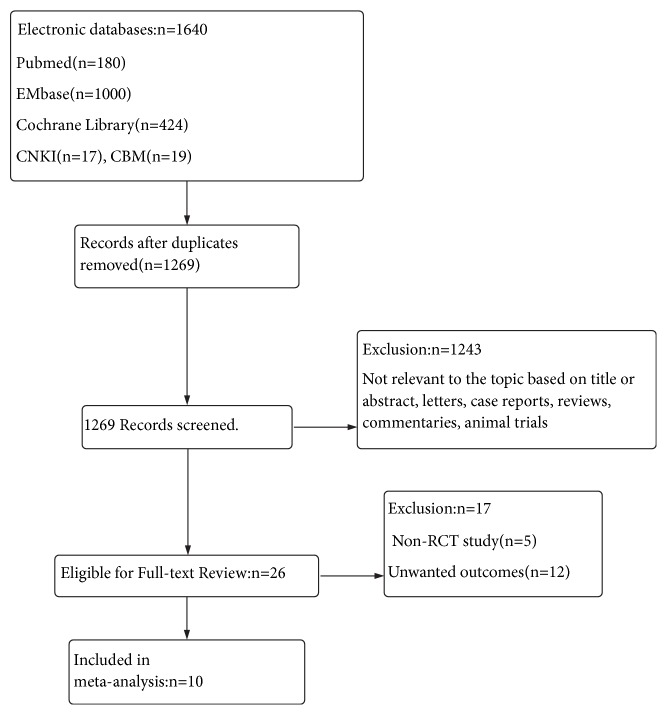
*PRISMA flow diagram.* Flow diagram demonstrating the process of article selection for systematic review and meta-analysis.

The electronic database search identified 1640 articles. A flow chart showing the identification of the RCTs for inclusion is shown in Figure 1. Of these 1269 articles, 10 RCTs met the selection criteria and were suitable for inclusion in this meta-analysis (Table 1) [15–24]. Among these studies, 586 patients were assigned to an aldosterone antagonists group, and 586 subjects were assigned to the control group.

**Figure 2:**
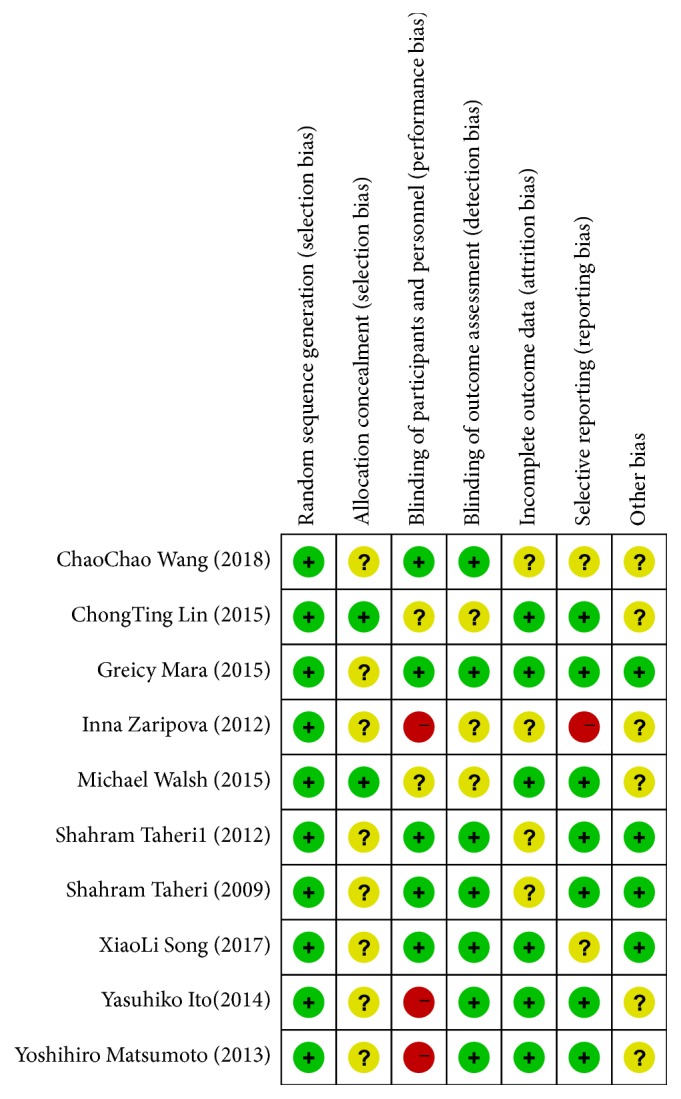
*Risk-of-bias summary.* Review of authors' judgments about each risk-of-bias item for each included study.

The author's judgments with regard to the risk of bias for each included study were assessed using the Cochrane's risk-of-bias tool shown in Figure 2. The risk of bias in the included studies was relatively moderate. Seven studies comparing aldosterone antagonists with placebo had lower overall risks of bias. The remaining three studies were open-labeled in which participants and personnel were not blinded, but the outcome assessors were blinded in two of the three studies.

### 3.2. Outcomes of Interest

#### 3.2.1. CCV Mortality and All-Cause Mortality

**Figure 3:**
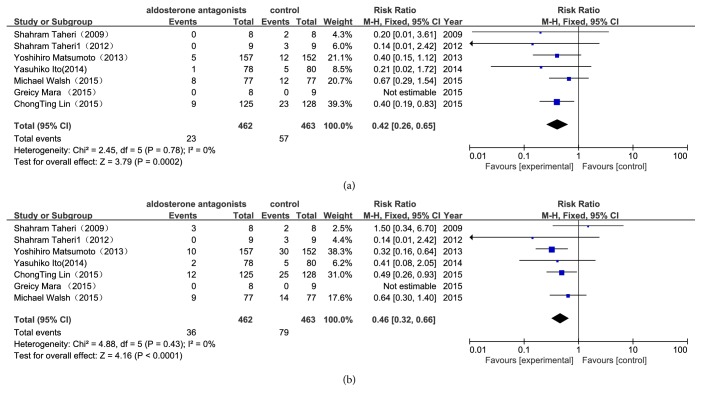
*Forest plots of the risk ratio for CCV mortality and all-cause mortality ((a) CCV mortality; (b) all-cause mortality).* The Chi-squared test is a measurement of heterogeneity. P<0.05 indicates significant heterogeneity. Squares = individual study point estimates. Horizontal lines = 95% CIs. Rhombus = summarized estimate and its 95% CI. Fixed: fixed effect model. Random: random effect model.

The results of the meta-analysis showed that of the 462 patients with aldosterone antagonist treatment, 23 reached cardiovascular-related death, which was significantly fewer than the 57 of the 463 patients assigned to the control group (RR 0.42, 95%CI 0.26-0.65, P=0.0002) (Figure 3(a)). The all-cause mortality in the aldosterone antagonist group was lower than in the control group (RR 0.46, 95%CI 0.32-0.66, P<0.0001) (Figure 3(b)). Clearly, the aldosterone antagonist treatment showed a statistically significant benefit in the reduction of CCV mortality and all-cause mortality.

#### 3.2.2. Left Ventricular Structure and Function

**Figure 4:**
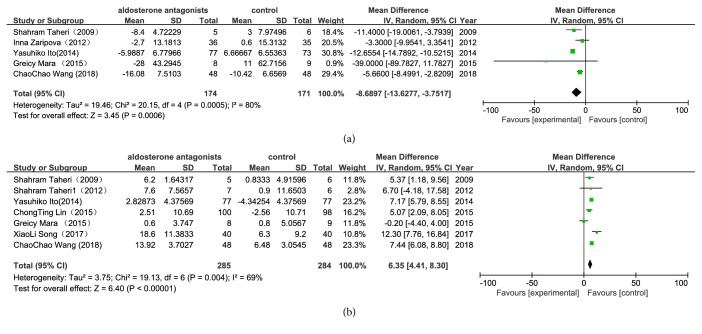
*Forest plots of the mean difference for the left ventricular mass index (LVMI) and the left ventricular ejection fraction (LVEF) ((a) LVMI; (b) LVEF).* The Chi-squared test is a measurement of heterogeneity. P<0.05 indicates significant heterogeneity. Squares = individual study point estimates. Horizontal lines = 95% CIs. Rhombus = summarized estimate and its 95% CI. Fixed: fixed effect model. Random: random effect model.

Among the 10 trials, only 5 trials recorded the LVMI from the baseline to the end of the study. The results showed that the additional aldosterone antagonist therapy was superior to the standard therapy with respect to LVMI (WMD -8.69g/m^2^, P=0.0006) (Figure 4(a)) through modification of the functional parameters of the left ventricle. Aldosterone antagonist therapy was similarly explored to improve LVEF (WMD 6.35%, P<0.00001) (Figure 4(b)). We found that the data collected were heterogeneous; we selected a random effect model for statistical analysis, and these models were also heterogeneous. The heterogeneity observed in our primary analysis may be explained by the trial designs, the dialysis method, the difference in the doses of aldosterone antagonists used, and the variation in the duration of treatment. The substitution of a fixed effect model for a random effect model did not change our initial qualitative interpretation of the pooled treatment effect on LVEF.

#### 3.2.3. Adverse Effects

**Figure 5:**
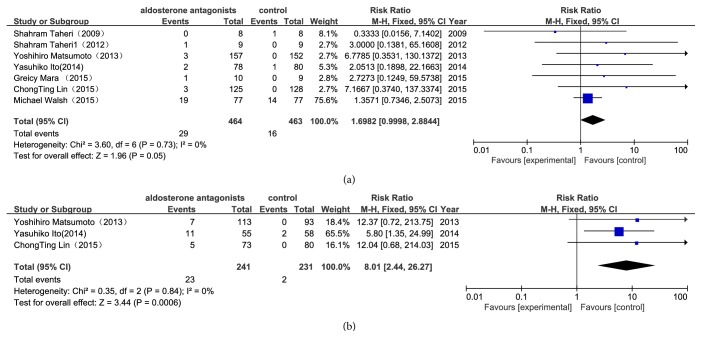
*Forest plots for adverse events. (a) Hyperkalemia; (b) nonphysiologic gynecomastia.* The Chi-squared test is a measurement of heterogeneity. P<0.05 indicates significant heterogeneity. Squares = individual study point estimates. Horizontal lines = 95% CIs. Rhombus = summarized estimate and its 95% CI. Fixed: fixed effect model. Random: random effect model.

As illustrated in Figure 5(a), compared with the control group, aldosterone antagonist treatment increased the occurrence of hyperkalemia, whereas this results showed no statistical significance (RR 1.70, 95%CI1-2.88, P=0.05). In the aldosterone antagonist group, there is a significant increase in the incidence of gynecomastia (RR 8.01, 95% CI2.44- 26.27, P=0.0006) (Figure 5(b)).

#### 3.2.4. Sensitivity Analysis

To evaluate the stability of our results, a sensitivity analysis was performed. No significant changes were detected between the previous and new results, with the latter pooled by the studies left when we deleted an individual study one at a time (S3). This result suggests that the association was convincible.

#### 3.2.5. Publication Bias

We carried out an assessment of the publication bias of the eligible studies. There was no obvious asymmetry in the funnel plot, and no evident publication bias was found with the P value of Egger's test (P =0.088) (S 4).

## 4. Discussion

Aldosterone antagonists play an important role in the treatment of severe heart failure patients [9]. We evaluated the efficacy and safety profile of adding aldosterone antagonists to the recommended standard treatment for dialysis patients. In the present meta-analysis, to produce reliable results, we used rigorous inclusion criteria and included only RCT studies. A total of 10 trials involving 1172 patients met our criteria and were enrolled in our meta-analysis. These moderate-to-high-quality studies indicated that aldosterone antagonists reduced the risk of mortality and improved cardiac function in patients undergoing maintenance dialysis.

In our findings, we demonstrated a reduction in CCV mortality and all-cause mortality in the aldosterone antagonists group. It is well known that the intensity of hemodialysis, blood pressure level, treatment with ACEI, or ARB is associated with cardiac outcomes. In our study, we may exclude the effect of the intensity of hemodialysis to cardiac outcomes as it was the same (about three times a week) between two groups. Most of the included studies show that the baseline blood pressure (BP) was similar between two groups; although there is no adequacy information, the researches declare no significant changes in BP level. And the proportion of ACEI or ARB use was similar between the two groups. Considering all of this, we could say that the addition of aldosterone antagonists could reduce the CCV mortality.

Cardiac function was also improved in the aldosterone antagonists group. Left ventricular mass (LVM) is a powerful independent predictor of cardiovascular morbidity and mortality [25]. Increased LVM has also been associated with coronary artery disease, all-cause mortality, and sudden death [26]. In recent analysis, beneficial effects were demonstrated in the changes of LV reverse remodeling and LV function. In our study, we found a -8.69 g/m^2^ reduction in the LVMI; although these results had some statistical heterogeneity. Aldosterone antagonists slowed the progression of LV remodeling, improved cardiac function, and reduced the occurrence of cardiac death.

Because of a relatively high heterogeneity in the LVMI and LVEF, we investigated the source of the heterogeneity. Meta-regression is typically performed to assess the factor that may have resulted in heterogeneity, but this was impossible in this meta-analysis because of the small number of trials included. Additionally, we attempted to conduct subgroup analyses according to the differences in race, dialysis method, duration of follow-up, and the difference in the doses of aldosterone antagonists used, but the subgroup analyses did not explain the heterogeneity observed in the outcomes. We substituted a random effect model for the fixed model, but we did not find changes in our initial qualitative interpretation of the pooled treatment effect on the LVEF and LVMI. Sensitivity analyses based on quality assessment did not alter the pooled results, and this result adds robustness to our main results.

This meta-analysis demonstrated that aldosterone antagonists may play a crucial role in dialysis patients. This treatment improves LV reverse modeling metabolism in cardiomyocytes and ultimately contributes to improvements in cardiac function and clinical symptoms.

While the safety of aldosterone antagonists is of considerable concern, the most commonly reported side effects were hyperkalemia and gynecomastia. In this meta-analysis, among patients receiving aldosterone antagonists, there was an increased occurrence of hyperkalemia compared with the control group, but this results showed no statistical significance. As our studied patients were undergoing chronic dialysis, the dialysis itself could remove the excessive potassium. Conversely, frequently potassium monitoring and strict restriction of potassium intake may be enough to prevent the recurrence of hyperkalemia. Another important adverse event was gynecomastia. Our analysis implied that the incidence of gynecomastia was significantly increased in the aldosterone antagonist group (9.5%) compared with the control group (0.87%). Actually, gynecomastia has previously been observed in patients who were treated with spironolactone [9]. Gynecomastia has been reported to occur in 10% of 1663 heart failure patients who received daily doses of spironolactone of 25 mg for 24 months [27]. The use of a selective aldosterone-receptor antagonist, such as eplerenone, may avoid the incidence of adverse effects like hyperkalemia, gynecomastia, and vaginal bleeding [28]. As studies of the effect of eplerenone in dialysis patients with respect to cardiac events are very limited, our study only included 1 trial of eplerenone, so we could not compare the adverse events between the spironolactone group and the eplerenone group. Further studies could estimate the effects of this treatment. However, the risk of gynecomastia could not be an argument against the use of spironolactone in patients undergoing chronic dialysis because spironolactone reduced the risk of CCV mortality. Overall, treatment with an aldosterone antagonist is feasible, although several adverse effects were observed.

Comparing our results with previously published articles, we found that treatment with an aldosterone antagonist for patients with acute myocardial infarction, heart failure, diabetic nephropathy, resistant hypertension, or other cardiovascular-related diseases has similar benefits and also results in similar adverse effects [29–32].

Although we could investigate certain positive effects in these trials, there were still some limitations. Firstly, only a small number of articles were included in this study, there are 3 out of 10 studies including less than 20 patients. Secondly, each article included people with different race, country, follow-up duration, and even different use of the drug doses, which could result in potential bias. Thus, these factors may have a potential impact on our results, and it should be updated by including more upcoming reports.

Further studies should focus on the following points. First, there was a need for further studies of the most suitable dosage and treatment duration for the aldosterone antagonist to observe benefits and adverse events. Second, although aldosterone antagonists were generally considered to reduce cardiac mortality, future studies are needed to determine the effects after long-term follow-up. Finally, in such future studies, the effect of aldosterone antagonists on hyperkalemia and nonphysiologic gynecomastia in the dialysis patients should also be given more attention. Some large-scale RCTs are currently underway to confirm the effect of aldosterone antagonists on mortality and cardiac function. The results of these studies are anticipated.

## 5. Conclusion

Our analysis indicated that treatment with aldosterone antagonists in chronic dialysis patients may reduce the risk of CCV mortality, improve cardiac function, and simultaneously decrease LVMI although several adverse effects were observed. Considering the prognostic importance of CCV in the dialysis population and the clear beneficial effect of aldosterone antagonists in this meta-analysis, high-quality randomized clinical trials need to be more actively undertaken.

## Figures and Tables

**Table 1:** Characteristics of all qualified studies included in the meta-analysis.

Study	Country	patients	N. of P.	Treatment	Drug control	Follow-up	Outcomes
Shahram Taheri (2009)	Iran	HD	16	Spironolactone 25mg three times a week	placebo	6 months	LVMI, LVEF, adverse events

Shahram Taheri (2012)	Iran	PD	18	Spironolactone 25mg every other day	placebo	6 months	LVEF, CCV mortality, all-cause mortality, adverse events

Inna Zaripova (2012)	Russian	HD	71	Spironolactone 25mg once daily	N/A	6 months	LVMI

Yoshihiro Matsumoto (2013)	Japan	HD	309	Spironolactone 25mg once daily	N/A	36 months	CCV mortality, all-casuse mortality, adverse events

Yasuhiko Ito (2014)	Japan	PD	158	Spironolactone 25mg once daily	N/A	24 months	LVMI, LVEF, all-cause mortality, adverse events

Greicy Mara (2015)	Brazil	HD	17	Spironolactone 12.5mg or 25mg once daily	placebo	6 months	LVMI, LVEF, SBP, DBP, aldosterone, adverse enents

Michael Walsh (2015)	Canada	HD	154	Eplerenone 50mg once daily	placebo	3months	CCV mortality, all-cause mortality, adverse events

ChongTing Lin (2015)	China	HD+PD	253	Spironolactone 25mg once daily	placebo	24 months	LVMI, CCV mortality, all-cause mortality, aldosterone, adverse events

XiaoLi Song (2017)	China	HD	80	Spironolactone 5mg once daily	placebo	12 months	LVEF

ChaoChao Wang (2018)	China	PD	96	Spironolactone 20mg once daily+ACEI/ARB	ACEI/ARB	12months	LVEF, LVMI

N. of P.: the number of patients; HD: hemodialysis; PD: peritoneal dialysis; N/A: no treatment.

## Supplementary Materials

Supplementary Materials S1 PRISMA checklist. S2 Search methods. S3 Sensitivity analysis. S4 Publish bias.
