# Supplementary material for: Aldosterone Antagonists Reduce the Risk of Cardiovascular Mortality in Dialysis Patients: A Meta-Analysis
Source: Evid Based Complement Alternat Med. 2019 Mar 3;2019:1925243. doi: 10.1155/2019/1925243 (PMC6421009; doi:10.1155/2019/1925243)
Supplement: Supplementary Materials — S1 PRISMA checklist. S2 Search methods. S3 Sensitivity analysis. S4 Publish bias. [file 1925243.f1.pdf]

S1 PRISMA checklist

Section/topic	#	Checklist item	Reported on page #
**TITLE**			
Title	1	Identify the report as a systematic review, meta-analysis, or both.	1
**ABSTRACT**			
Structured summary	2	Provide a structured summary including, as applicable: background; objectives; data sources; study eligibility criteria, participants, and interventions; study appraisal and synthesis methods; results; limitations; conclusions and implications of key findings; systematic review registration number.	1
**INTRODUCTION**			
Rationale	3	Describe the rationale for the review in the context of what is already known.	1,2
Objectives	4	Provide an explicit statement of questions being addressed with reference to participants, interventions, comparisons, outcomes, and study design (PICOS).	2
**METHODS**			
Protocol and registration	5	Indicate if a review protocol exists, if and where it can be accessed (e.g., Web address), and, if available, provide registration information including registration number.	N/A
Eligibility criteria	6	Specify study characteristics (e.g., PICOS, length of follow-up) and report characteristics (e.g., years considered, language, publication status) used as criteria for eligibility, giving rationale.	3
Information sources	7	Describe all information sources (e.g., databases with dates of coverage, contact with study authors to identify additional studies) in the search and date last searched.	2,3
Search	8	Present full electronic search strategy for at least one database, including any limits used, such that it could be repeated.	3
Study selection	9	State the process for selecting studies (i.e., screening, eligibility, included in systematic review, and, if applicable, included in the meta-analysis).	3
Data collection process	10	Describe method of data extraction from reports (e.g., piloted forms, independently, in duplicate) and any processes for obtaining and confirming data from investigators.	3,4
Data items	11	List and define all variables for which data were sought (e.g., PICOS, funding sources) and any assumptions and simplifications made.	3
Risk of bias in individual studies	12	Describe methods used for assessing risk of bias of individual studies (including specification of whether this was done at the study or outcome level), and how this information is to be used in any data synthesis.	4
Summary measures	13	State the principal summary measures (e.g., risk ratio, difference in means).	4
Synthesis of results	14	Describe the methods of handling data and combining results of studies, if done, including measures of consistency (e.g., I^2) for each meta-analysis.	4

Section/topic	#	Checklist item	Reported on page #
Risk of bias across studies	15	Specify any assessment of risk of bias that may affect the cumulative evidence (e.g., publication bias, selective reporting within studies).	4
Additional analyses	16	Describe methods of additional analyses (e.g., sensitivity or subgroup analyses, meta-regression), if done, indicating which were pre-specified.	4
**RESULTS**			
Study selection	17	Give numbers of studies screened, assessed for eligibility, and included in the review, with reasons for exclusions at each stage, ideally with a flow diagram.	4
Study characteristics	18	For each study, present characteristics for which data were extracted (e.g., study size, PICOS, follow-up period) and provide the citations.	5
Risk of bias within studies	19	Present data on risk of bias of each study and, if available, any outcome level assessment (see item 12).	4,5
Results of individual studies	20	For all outcomes considered (benefits or harms), present, for each study: (a) simple summary data for each intervention group (b) effect estimates and confidence intervals, ideally with a forest plot.	5,6
Synthesis of results	21	Present results of each meta-analysis done, including confidence intervals and measures of consistency.	5,6
Risk of bias across studies	22	Present results of any assessment of risk of bias across studies (see Item 15).	6,7
Additional analysis	23	Give results of additional analyses, if done (e.g., sensitivity or subgroup analyses, meta-regression [see Item 16]).	6
**DISCUSSION**			
Summary of evidence	24	Summarize the main findings including the strength of evidence for each main outcome; consider their relevance to key groups (e.g., healthcare providers, users, and policy makers).	7,8
Limitations	25	Discuss limitations at study and outcome level (e.g., risk of bias), and at review-level (e.g., incomplete retrieval of identified research, reporting bias).	8
Conclusions	26	Provide a general interpretation of the results in the context of other evidence, and implications for future research.	8
**FUNDING**			
Funding	27	Describe sources of funding for the systematic review and other support (e.g., supply of data); role of funders for the systematic review.	N/A

From: Moher D, Liberati A, Tetzlaff J, Altman DG, The PRISMA Group (2009). Preferred Reporting Items for Systematic Reviews and Meta-Analyses: The PRISMA Statement. PLoS Med 6(6): e1000097. doi:10.1371/journal.pmed1000097

For more information, visit: **www.prisma-statement.org**.

S2 Search methods

Pubmed:
(((renal dialysis) OR (renal insuffic*) OR (kidney failure) OR (peritoneal dialysis) OR hemodialysis) OR (renal dialysis[MeSH] OR (Renal Insufficiency[MeSH] OR kidney failure[MeSH] OR peritoneal dialysis[MeSH] OR Hemodialysis[MeSH])) AND ((spironolactone OR spirolactone OR androsterone OR eplerenone OR (mineralocorticoid receptor antagonist) OR (aldosterone receptor antagonist)) OR (mineralocorticoid receptor antagonists[MeSH] OR spironolactone[MeSH])) AND (randomized controlled trial[pt] OR controlled clinical trial[pt] OR randomized[tiab] OR placebo[tiab] OR clinical trials as topic[mesh:noexp] OR randomly[tiab] OR trial[ti]) NOT (animals[mh] NOT humans[mh])

EMBASE:
#1 'renal dialysis' OR 'renal insufficiency' OR 'kidney failure' OR 'peritoneal dialysis' OR hemodialy*
#2 'dialysis'/exp OR 'hemodialysis'/exp OR 'continuous ambulatory peritoneal dialysis'/exp OR 'end stage renal disease'/exp OR 'kidney failure'/exp
#3 spironolactone* OR spirolactone* OR antisterone* OR eplerenone* OR 'mineralocorticoid antagonist'
#4 'mineralocorticoid antagonist'/exp OR 'spironolactone' OR 'eplereone'
#5 #3 OR #4
#6 #1 OR #2
#7 #5 AND #6
#8 random* OR factorial* OR crossover* OR placebo*
#9 'crossover-procedure'/exp OR 'double-blind procedure'/exp OR 'randomized controlled trial'/exp OR 'single blind procedure'
#10 #8 OR #9
#11 #7 AND #10

Cochrane library:
#1MeSH descriptor: [Renal Dialysis] explode all trees
#2MeSH descriptor: [Renal Insufficiency] explode all trees
#3MeSH descriptor: [Peritoneal Dialysis] explode all trees
#4#1 or #2 or #3
#5((renal dialysis) or (renal insufficiency) or (kidney failure) or (peritoneal dialysis) or hemodialy*)
#6#4 or #5
#7MeSH descriptor: [Mineralocorticoid Receptor Antagonists] explode all trees
#8MeSH descriptor: [Spironolactone] explode all trees
#9(spironolactone or spirolactone or antisterone* or eplerenone* or (mineralocorticoid receptor antagonist) or (aldosterone receptor antagonist))
#10#7 or #8 or #9
#11#6 and #10

S3 Sensitivity analysis

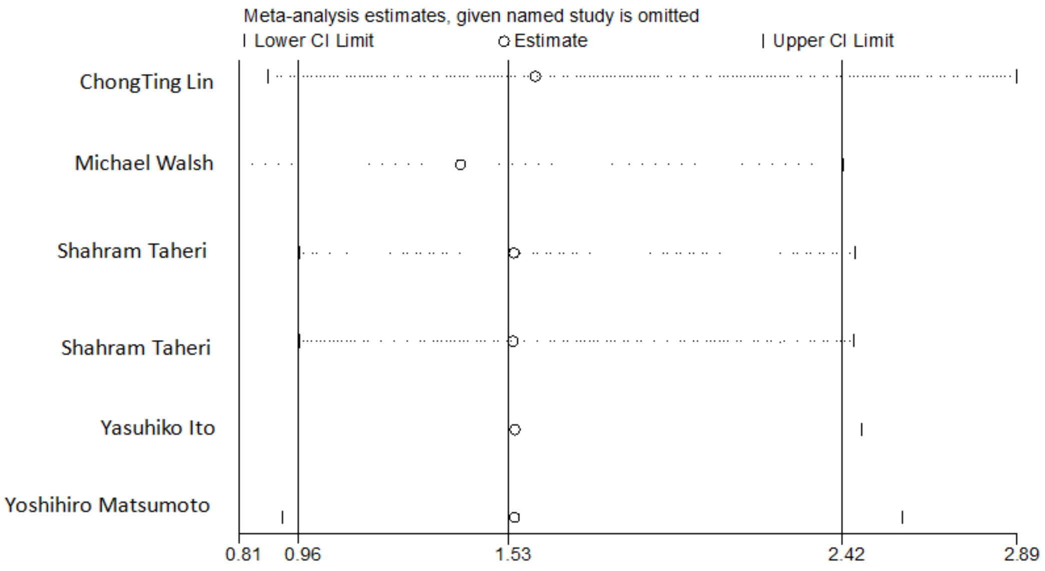

S3. Sensitivity analysis of CCV mortality

S4 Publish bias

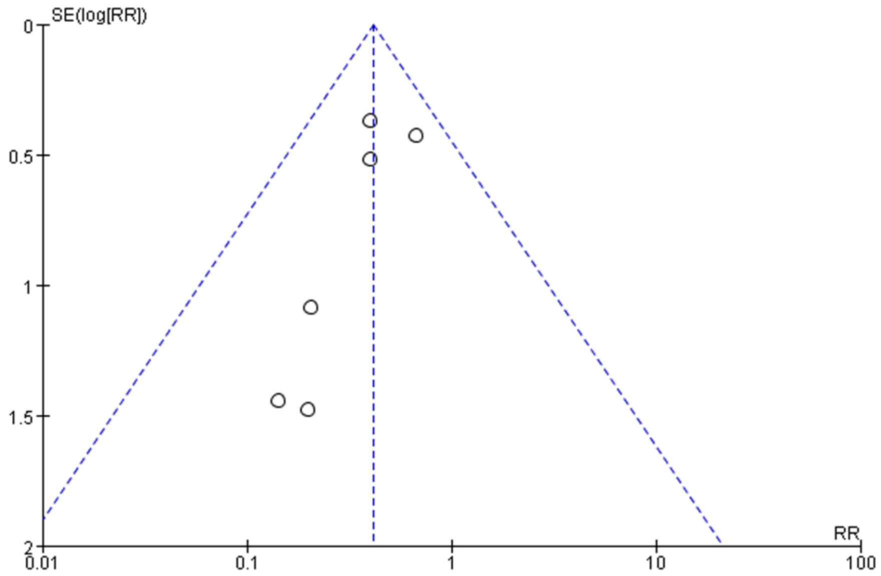

S4A Funnel plot of the CCV mortality.

Egger's test for small-study effects:
Regress standard normal deviate of intervention
effect estimate against its standard error

Number of studies = 6 Root MSE = .5172

Std_Eff	Coef.	Std. Err.	t	P>|t|	[95% Conf.	Interval]
slope	-.3175278	.2604886	-1.22	0.290	-1.04076	.4057045
bias	-1.019233	.453403	-2.25	0.088	-2.278081	.239616

Test of H0: no small-study effects P = 0.088

S4B Egger's test of CCV mortality
